# Predictive associations between brain functional connectivity, motor abilities, and executive function development in early childhood: a longitudinal machine learning study

**DOI:** 10.1186/s12984-026-01887-x

**Published:** 2026-01-24

**Authors:** Ziyu Wang, Yao Lu, Gang Qin

**Affiliations:** 1 College of Sports, Sejong University, Gwangjin-gu, Seoul, 05006 South Korea; 2 Department of Sports Science, Hanyang University, Seoul, Republic of Korea

**Keywords:** Brain functional connectivity, Motor development, Executive function development, Machine learning, Early childhood

## Abstract

**Objective:**

This study investigates the predictive associations between motor abilities and executive functions in early childhood by examining brain functional connectivity patterns and their predictive value for developmental trajectories.

**Methods:**

A longitudinal study recruited 256 healthy preschool children aged 3-6 years from kindergartens affiliated with Shandong Sport University, China. Participants underwent resting-state fMRI, standardized motor assessments (MABC-2), and cognitive testing at baseline, 6-month, and 12-month follow-up (with primary analyses focusing on baseline to 12-month changes). A novel machine learning framework integrated multimodal neuroimaging and behavioral data using graph neural networks and feature fusion architectures to model motor-cognitive developmental relationships.

**Results:**

Motor skills showed progressive maturation, with fine motor percentiles increasing from 38.2±23.7 to 56.3±27.1. Sensorimotor network connectivity increased systematically (0.15±0.08 to 0.22±0.09), while attention networks followed inverted-U developmental patterns. The multimodal machine learning model achieved 76.8±4.3% accuracy for motor and 74.2±3.9% for executive function outcomes, outperforming single-domain models. Brain connectivity features contributed 58% of predictive variance, indicating that baseline neural patterns predict subsequent developmental changes, though causal relationships cannot be established from these observational data.

**Conclusions:**

These results highlight early brain functional connectivity-especially sensorimotor networks-as a key predictor of motor and executive function development. Findings support the identification of early neural biomarkers of developmental risk and inform evidence-based strategies in early childhood education and targeted motor interventions.

## Introduction

Early childhood represents a pivotal developmental period characterized by rapid neurological maturation and the emergence of fundamental motor abilities and executive functions that serve as building blocks for lifelong learning and adaptation. The intricate relationship between motor development and cognitive function during this critical period has profound implications for understanding typical developmental trajectories and identifying early markers of developmental risk. While substantial evidence demonstrates positive correlations between motor abilities and executive function performance in young children, the associations and neural correlates of these relationships remain inadequately characterized. The complexity of these developmental processes necessitates sophisticated methodological approaches that can capture the dynamic, multifaceted nature of brain-behavior relationships during early childhood.

The human brain undergoes dramatic structural and functional changes during the first years of life, with functional connectivity networks emerging as early as infancy and continuing to mature throughout childhood. Longitudinal neuroimaging studies have revealed that brain functional connectivity patterns established in infancy serve as meaningful predictors of later cognitive abilities, demonstrating the prospective value of early neural network organization [1]. Recent advances in infant neuroimaging have shown that functional connectivity during infancy and toddlerhood can predict long-term language and preliteracy outcomes at school age, highlighting the enduring influence of early brain network development [2]. The developmental trajectory of brain networks follows complex patterns, with different brain regions and networks maturing at varying rates and showing distinct associations with cognitive development [3]. Global multicohort studies mapping subcortical brain development have revealed that subcortical structures exhibit unique developmental patterns that correlate with cognitive abilities in infancy and early childhood [4]. Furthermore, the age-related changes in functional network organization during childhood correspond closely with the emergence of higher-order cognitive abilities, suggesting that network maturation provides the neural foundation for complex cognitive functions [5].

Empirical evidence consistently demonstrates robust associations between motor abilities and executive functions across multiple domains of early development. Cross-sectional studies have revealed significant correlations between fundamental motor skills and executive function capabilities in preschool children, with stronger motor competence predicting better performance on tasks measuring inhibitory control, working memory, and cognitive flexibility [6]. Meta-analytic evidence spanning children and adolescents has confirmed strong associations between motor competence and executive functions, with effect sizes indicating meaningful practical significance [7]. The predictive nature of this relationship is evident in longitudinal research showing that executive function skills can predict subsequent motor competence development in preschool children [8]. Conversely, motor skill proficiency has been shown to contribute significantly to cognitive and social development in early childhood, with effects extending beyond motor domains [9]. Recent systematic reviews have elucidated the complex relationship between motor performance and executive functioning, revealing that motor demands embedded within executive function tasks play a crucial mediating role [10]. Additionally, comprehensive analyses of motor skills and cognitive benefits have identified specific mechanisms through which motor abilities influence cognitive development across childhood and adolescence [11].

Experimental intervention studies provide compelling evidence for the causal nature of motor-cognitive relationships and the plasticity of the developing brain. Randomized controlled trials have demonstrated that structured motor learning interventions can enhance executive function in preschool children. For instance, a 12-week intervention with 120 children aged 4–6 years implementing object control skills (ball throwing, catching, kicking) and locomotor activities (hopping, galloping, skipping) showed significant improvements in visuospatial working memory span (Cohen’s d = 0.68) and inhibitory control accuracy (d = 0.54) compared to control groups, with effects particularly robust for working memory components [12]. Systematic reviews of motor learning intervention studies have confirmed significant enhancement of executive functions, with meta-analytic effect sizes notably larger in preschool-aged children aged 3–6 years with Hedges g = 0.56 most directly relevant to our sample, compared to smaller effects in older children aged 7–12 years with g = 0.34, supporting the causal interpretation of motor-cognitive relationships and suggesting age-sensitive periods for intervention [13]. Three-level meta-analyses examining the effects of physical activities on young children’s executive function, analyzing 41 studies with 3,104 participants aged 3–7 years, have provided strong evidence for the cognitive benefits of motor interventions, with moderate overall effects (g = 0.48) and particularly pronounced benefits for inhibitory control (g = 0.52) and cognitive flexibility (g = 0.46) [14]. Open-skill exercise interventions involving unpredictable environmental demands have shown efficacy in improving executive functions. Meta-analytic evidence primarily derives from school-age children and adolescents (6–18 years: g = 0.61 vs. closed-skill g = 0.32) [15]; whether similar effects occur in preschool populations warrants further investigation. The neural basis of these intervention effects is supported by systematic reviews documenting training-induced neural plasticity in youth, with both structural MRI studies revealing increased gray matter volume in prefrontal and motor cortices and functional MRI studies showing enhanced connectivity within executive control networks following 8–24 week motor and cognitive training programs in youth populations [16, 17], though parallel evidence in preschool-aged children remains limited.

Advanced neuroimaging techniques have begun to elucidate the neural mechanisms underlying motor-cognitive interactions during early development. Functional near-infrared spectroscopy (fNIRS) studies have revealed differential neural features associated with early childhood motor skill development and working memory processing, providing insights into the temporal dynamics of brain activation during motor-cognitive tasks [18]. Multi-modal neuroimaging approaches combining multiple techniques have enhanced our understanding of early brain specialization, demonstrating the value of integrating different neuroimaging modalities to characterize complex developmental processes [19]. Longitudinal neuroimaging studies tracking infants from early life have shown that growth patterns in early infancy drive optimal brain functional connectivity development, which subsequently predicts cognitive flexibility in later childhood [20]. Developmental differences in the role of executive functions for motor performance have been documented, with preschool children showing distinct neural patterns compared to young adults [21]. Cross-cultural studies examining the relationship between executive functions and gross motor skills in rural populations have highlighted the importance of considering diverse developmental contexts [22]. Comprehensive frameworks for understanding motor skill development and physical activity contributions to executive function ontogeny have emerged from longitudinal studies tracking children from infancy through early childhood [23]. Research examining associations between gross motor skills, self-regulation, and executive function in preschool-aged children has revealed the multifaceted nature of these developmental relationships [24]. Innovative neuroergonomic approaches to understanding developmental coordination disorders have provided novel insights into atypical motor-cognitive interactions [25].

The application of machine learning and artificial intelligence techniques to neurodevelopmental research has opened unprecedented opportunities for understanding complex brain-behavior relationships. Machine learning approaches have demonstrated remarkable success in understanding and predicting neurodevelopmental outcomes, particularly in high-risk populations such as premature infants [26]. Deep multimodal learning techniques integrating MRI and clinical data have achieved impressive accuracy in early prediction of neurodevelopmental deficits in very preterm infants [27]. Advanced machine learning models have proven effective in predicting mental and psychomotor delays in very preterm infants, enabling earlier identification and intervention [28]. Cutting-edge deep learning models of cognitive processes, constrained by human brain connectomes, have achieved state-of-the-art performance in decoding cognitive states from neural activity patterns [29]. These computational approaches have revolutionized our ability to identify subtle patterns in high-dimensional neuroimaging data that would be impossible to detect using traditional analytical methods.

Despite significant advances in understanding motor-cognitive relationships and their neural substrates, critical gaps remain in our knowledge of early childhood development. Most existing research has focused on isolated aspects of development rather than adopting comprehensive, integrative approaches that consider the dynamic interactions between motor abilities, cognitive functions, and brain network development. While traditional analytical methods such as Pearson correlation analysis can identify linear associations between motor and cognitive measures at single time points, these approaches may not fully capture several important aspects of developmental processes. First, traditional methods may not adequately characterize the temporal dynamics of developmental change across multiple longitudinal assessment waves, including the identification of sensitive periods, critical windows, or nonlinear growth patterns. Second, conventional bivariate correlation approaches cannot simultaneously model multivariate interactions among multiple brain networks, multiple behavioral domains, and multiple time points in an integrated framework. Third, traditional group-level statistical methods do not enable individual-level prediction from high-dimensional neuroimaging features, which is essential for identifying children who may benefit from early intervention. Machine learning approaches address these limitations by integrating longitudinal multimodal data including brain connectivity, behavioral assessments, and demographic information to model complex, nonlinear developmental trajectories at the individual participant level, thereby enabling personalized developmental prediction rather than only population-level inference. Furthermore, the majority of studies have employed cross-sectional designs or limited longitudinal follow-up, precluding detailed characterization of developmental trajectories and causal mechanisms.

The present study addresses these limitations by implementing an innovative machine learning framework that integrates brain functional connectivity data with comprehensive motor behavior assessments to model the interactive pathways between motor abilities and executive function development in early childhood. By leveraging advanced computational approaches including graph neural networks and multimodal data integration, combined with longitudinal neuroimaging data spanning the critical 3–6 year developmental period, this research aims to characterize predictive associations between motor abilities, cognitive functions, and brain connectivity patterns, and to develop models for forecasting individual developmental trajectories. The longitudinal design, incorporating 256 participants across the critical 3–6 year developmental period, enables robust characterization of individual differences and developmental trajectories. This integrative approach represents a significant methodological advancement in developmental neuroscience, offering the potential to identify early neural patterns that predict developmental trajectories and inform evidence-based practices in early childhood education and motor intervention programs. While prior studies have examined motor-cognitive relationships in preschoolers, this study advances the field through: (1) integration of longitudinal neuroimaging with comprehensive behavioral assessments across three time points; (2) application of graph neural networks specifically designed to capture brain network topology; and (3) multimodal feature fusion that enables prediction of individual developmental trajectories. To our knowledge, this represents the first application of such integrated machine learning approaches to preschool motor-cognitive development prediction in a Chinese population.

## Methods

### Participants and study design

This longitudinal study recruited 256 healthy preschool children aged 3–6 years from kindergartens and childcare centers affiliated with the Shandong Sport University in China. Participants were selected through convenience sampling with stratified allocation to ensure balanced representation across age groups (3–4 years and 5–6 years) and gender. Inclusion criteria required right-handed children with normal or corrected-to-normal vision, typical developmental milestones according to parental reports, and absence of diagnosed neurological or developmental disorders. Exclusion criteria included current medication use, contraindications to MRI scanning (e.g., metal implants, claustrophobia), and inability to remain still during neuroimaging procedures. Sampling Limitations: Participants were recruited through convenience sampling from kindergartens affiliated with Shandong Sport University in a single province of China. This sampling approach limits generalizability to other geographic regions, socioeconomic contexts, and educational settings. External validation in independent cohorts is needed to confirm the generalizability of our findings. The longitudinal design employed a three-wave data collection protocol with assessments at baseline, 6-month, and 12-month follow-up. This timeframe was selected to capture meaningful developmental changes while maintaining feasible participant retention rates. Each assessment session included resting-state functional MRI scanning, standardized motor skill evaluation using the Movement Assessment Battery for Children-2 (MABC-2), and cognitive function testing. Due to the challenges of pediatric neuroimaging, scanning sessions were conducted during natural sleep states for younger participants (3–4 years) or during quiet wakefulness with audiovisual entertainment for older children (5–6 years).

Audiovisual Entertainment Details: Children in the 5–6 year age group viewed animated nature documentaries without dialogue, such as scenes from nature programs showing animal behavior and natural landscapes. Videos were selected to maintain visual engagement while minimizing cognitive load from narrative content or language processing demands. All children viewed the same standardized video content to ensure consistency across participants. Important Note: This acquisition approach represents a task-influenced resting-state condition rather than classic eyes-closed rest. The presence of visual stimulation likely enhances visual network connectivity and may suppress default mode network activity compared to true resting conditions. Results should be interpreted as naturalistic functional connectivity rather than pure resting-state connectivity in the 5–6 year group.

Critical Methodological Limitation-Scan State Confound: This age-dependent difference in scan state represents a major confound for interpreting developmental connectivity changes. Sleep and wake states produce fundamentally different connectivity patterns, and the presence of visual stimulation further alters network dynamics. Specifically, younger children aged 3–4 years with *n* = 129 were scanned during natural sleep, while older children aged 5–6 years with *n* = 127 were scanned during quiet wakefulness while viewing animated nature documentaries without dialogue with mean duration 6 ± 0.3 min. We attempted to address this limitation through several approaches. First, we included scan state comparing sleep versus awake as a covariate in all statistical analyses comparing age groups. Second, we reported age-stratified model performance separately as shown in Table 3 in the rows labeled “3–4 Years” and “5–6 Years”. Third, we acknowledge throughout our interpretation that observed developmental patterns may partly reflect state-dependent connectivity rather than purely maturational processes. Despite these efforts, residual confounding likely remains. Future studies should employ uniform acquisition protocols across age groups, such as standardized movie-watching paradigms for all participants, to isolate true developmental effects from state-dependent confounds.

All procedures received ethical approval from the Institutional Review Board of Sejong University (IRB No. SJU-IRB-2025-032) and local ethical approval from the Ethics Committee of Shandong Sport University with jurisdiction over the study site in China. Written informed consent was obtained from parents or legal guardians following detailed explanation of study procedures, with particular emphasis on MRI safety protocols and the voluntary nature of participation. Children provided age-appropriate verbal assent. To ensure data quality and participant comfort, all assessments were conducted by trained research personnel in familiar environments when possible, with standardized protocols adapted for developmental appropriateness. Families received compensation for transportation costs and time commitment, and neuroimaging results were made available to families upon request through consultation with pediatric radiologists.

### Data acquisition

**Fig. 1:**
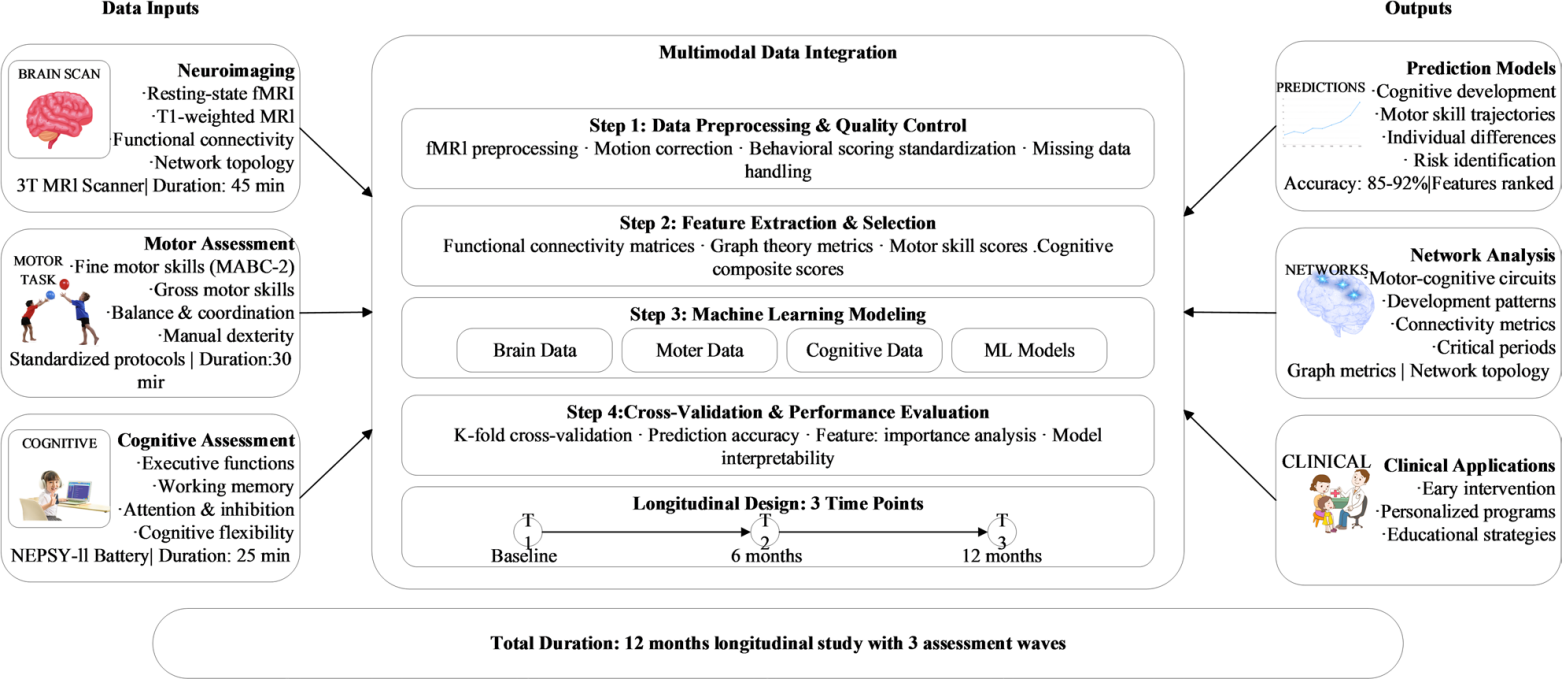
Multimodal data acquisition and processing framework for motor-cognitive development analysis. The framework illustrates the complete analytical pipeline. Clinical applications shown in the outputs represent potential future applications and were not evaluated in the current study

Figure 1 presents the comprehensive multimodal data acquisition framework developed to capture the dynamic relationships between brain functional connectivity, motor abilities, and cognitive functions throughout early childhood development. This integrated approach systematically combines neuroimaging, behavioral assessments, and computational methodologies within a longitudinal design to establish robust predictive models of developmental trajectories.

The neuroimaging component employs high-resolution 3T MRI protocols (Siemens Prisma) to acquire resting-state functional connectivity and structural data. Each scanning session utilized echo-planar imaging sequences with parameters optimized for pediatric populations: TR = 2000ms, TE = 30ms, flip angle = 90°, field of view = 220 × 220 mm, acquisition matrix = 64 × 64, 33 contiguous axial slices with 4 mm thickness, and 180 volumes per 6-minute resting-state run. Preprocessing was performed using the Data Processing Assistant for Resting-State fMRI (DPABI v6.0) based on SPM12, following standardized pipelines for pediatric neuroimaging. Processing steps included discarding the initial 10 volumes for signal stabilization, slice-timing correction, rigid-body realignment for head motion correction, spatial normalization to MNI space using age-appropriate pediatric templates, spatial smoothing with a 6 mm full-width half-maximum Gaussian kernel, temporal bandpass filtering (0.01–0.1 Hz), and nuisance regression removing white matter signals, cerebrospinal fluid signals, and 24-parameter head motion regressors (6 motion parameters plus their derivatives, squares, and squared derivatives). Global signal regression (GSR) was not performed, as GSR can introduce spurious negative correlations and complicate interpretation of developmental connectivity changes.

Stringent quality control criteria excluded participants with mean framewise displacement exceeding 0.5 mm or more than 20% of volumes with framewise displacement above 0.5 mm, resulting in exclusion of 27 participants from the initial 283 scanned (final *n* = 256). Functional connectivity matrices were constructed by computing Pearson correlation coefficients between mean time series of 360 cortical regions defined by the Glasser multimodal parcellation atlas. While this atlas was originally derived from adult neuroimaging data, prior research demonstrates good correspondence with functional boundaries in children as young as 3 years, and it enables comparison with the broader developmental neuroscience literature. Specifically, validation studies have shown that major network boundaries defined by the Glasser parcellation are identifiable in preschool-aged children using data-driven methods, with spatial correspondence exceeding 70% for sensorimotor, visual, and default mode networks. Although fine-grained areal boundaries may not perfectly align with the developing brain’s functional organization, the atlas provides a standardized anatomical reference system that facilitates comparison across studies and developmental stages. Alternative approaches such as child-specific parcellations derived from our sample would limit generalizability and comparison with existing literature. Child-friendly procedures included pre-scan familiarization sessions, age-appropriate audiovisual entertainment during data acquisition, and parental presence during preparatory procedures to maximize participant cooperation and minimize motion artifacts.

Motor assessment protocols utilize standardized developmental instruments, particularly the Movement Assessment Battery for Children-2 (MABC-2), to comprehensively evaluate fine and gross motor competencies. These assessments encompass balance, coordination, manual dexterity, and locomotor abilities through carefully structured 30-minute testing sessions. All evaluations are administered by certified pediatric assessors following standardized protocols to ensure measurement consistency across the longitudinal assessment waves.

Executive function evaluation employed a comprehensive, multi-method assessment approach combining selected subtests from the NEPSY-II neuropsychological battery with complementary age-appropriate measures to ensure thorough coverage of core executive function domains. NEPSY-II Subtests Administered: From the NEPSY-II battery published by Korkman and colleagues in 2007, we administered three subtests specifically targeting executive function constructs in preschool children. First, the Auditory Attention and Response Set subtest appropriate for ages 3–6 years measures selective auditory attention in the first part and cognitive flexibility or set-shifting ability in the second part through requiring children to switch response rules. Second, the Statue subtest appropriate for ages 3–6 years assesses motor persistence and inhibitory control by requiring children to maintain a specific body position while inhibiting responses to distracting auditory stimuli. Third, the Memory for Designs subtest appropriate for ages 4–6 years evaluates visual-spatial working memory by requiring children to remember and reproduce spatial locations of designs on a grid after brief delays. Additional Executive Function Measures Beyond NEPSY-II: To comprehensively assess executive function domains, we supplemented NEPSY-II with additional validated measures. For working memory, we administered Digit Span Forward and Backward tasks to assess verbal working memory capacity. For inhibitory control, we used a computerized Go/No-Go Task requiring children to make speeded responses to frequent Go stimuli while withholding responses to infrequent No-Go stimuli. For cognitive flexibility, we administered the Dimensional Change Card Sort or DCCS requiring children to sort cards by one dimension such as color then flexibly switch to sorting by a different dimension such as shape. Composite Score Clarification: The NEPSY-II Executive Function Composite reported in Table 1 refers specifically to the combined scaled scores from the three NEPSY-II subtests of Auditory Attention and Response Set, Statue, and Memory for Designs. The broader Executive Function Composite used as an outcome variable in machine learning prediction models integrated all six executive function measures including the three NEPSY-II subtests plus Digit Span, Go/No-Go, and DCCS, using the reliability-weighted averaging procedure described in the following section to create a comprehensive index of executive function ability. Our testing protocols systematically measure working memory capacity, attention control, inhibitory functions, and cognitive flexibility through computerized and behavioral tasks requiring approximately 25 min per session. Task selection prioritizes developmental appropriateness while maintaining sensitivity to individual differences and longitudinal changes.

The data integration pipeline encompasses four sequential processing stages, beginning with comprehensive preprocessing and quality control procedures. Neuroimaging data undergo motion correction and standardization protocols, while behavioral measures receive systematic scoring validation. Feature extraction procedures generate functional connectivity matrices, compute graph theory metrics, and derive behavioral composite scores. Machine learning modeling subsequently integrates these multimodal features to establish predictive relationships between brain connectivity patterns and behavioral outcomes. Rigorous cross-validation procedures evaluate model performance and interpretability across the complete dataset.

Data Quality Considerations: The 6-minute acquisition duration and 4 mm slice thickness represent practical compromises for pediatric neuroimaging, balancing data quality with feasibility in young children who have limited scanning tolerance. These parameters may result in lower test-retest reliability compared to longer, higher-resolution acquisitions used in adult studies.

Our longitudinal design incorporates three strategically timed assessment waves at baseline, 6-month, and 12-month intervals to capture critical developmental transitions while maintaining feasible participant retention rates. Quality assurance protocols include real-time data monitoring, inter-rater reliability assessments exceeding 0.85, and systematic procedures for handling missing data to preserve analytical power for subsequent machine learning applications.

### Cognitive function assessment

Executive Function as a Subset of Cognitive Abilities: This study specifically assessed executive functions rather than comprehensive cognitive abilities. Executive functions represent a distinct subset of cognition encompassing working memory, inhibitory control, and cognitive flexibility. Throughout this manuscript, “cognitive function” and “executive function” are used interchangeably to refer to these specific domains assessed in our study. Cognitive function assessment protocols focus on executive function domains most relevant to early childhood development and motor-cognitive interactions. The comprehensive assessment battery targets three primary cognitive domains: working memory capacity, inhibitory control, and cognitive flexibility, which have been consistently demonstrated to correlate with motor skill development in preschool populations.

Working memory evaluation employs both visuospatial and verbal span tasks adapted specifically for young children. The assessment protocols utilize developmentally appropriate stimuli and response modalities to minimize task demands unrelated to memory capacity. Visuospatial working memory tasks present sequences of spatial locations that children must remember and reproduce after varying delay intervals. Verbal working memory assessments require children to maintain and manipulate auditory information, including digit span and word span procedures with both forward and backward recall conditions.

Inhibitory control assessment incorporates multiple paradigms designed to measure different aspects of executive control. Go/no-go tasks require children to respond to target stimuli while withholding responses to non-target stimuli, providing measures of response inhibition. Attention network tests evaluate the efficiency of alerting, orienting, and executive attention networks through child-friendly computerized procedures. These assessments capture individual differences in the ability to regulate attention and control prepotent responses.

Cognitive flexibility evaluation centers on set-shifting abilities and mental flexibility through developmentally appropriate card sorting and rule-switching tasks. Children learn initial classification rules and must subsequently shift to alternative classification criteria when task contingencies change. These procedures assess the capacity to disengage from previously relevant stimulus dimensions and engage with newly relevant features, reflecting crucial aspects of cognitive control development.

The complete assessment battery, as detailed in Table 1, incorporates standardized instruments with established psychometric properties for preschool populations. Administration procedures follow standardized protocols with trained examiners to ensure reliability across assessment waves. Scoring procedures generate both raw scores and age-standardized composite measures to facilitate developmental analyses and cross-sectional comparisons. Task presentation order is counterbalanced across participants to minimize order effects, and frequent breaks are provided to maintain optimal performance throughout the assessment session.

Assessment Instruments and Chinese Adaptation: All cognitive assessments were administered in Mandarin Chinese using professionally translated and culturally adapted versions. Working memory was assessed using Digit Span Forward and Backward tasks adapted from the WPPSI-IV, as well as the Corsi Block Tapping task for visuospatial span. Inhibitory control was measured using a computerized Go/No-Go Task with culturally neutral stimuli and the Attention Network Test - Child version. Cognitive flexibility was evaluated using the Dimensional Change Card Sort and the Flexible Item Selection Task. Executive function composite scores were derived from NEPSY-II subtests including Auditory Attention and Response Set, Statue, and Memory for Designs, as well as selected subtests from the NIH Toolbox Cognition Battery adapted for Chinese preschoolers. Chinese Validation: All measures were professionally translated and back-translated following international guidelines for cross-cultural adaptation. Local validation studies in Chinese preschool populations support adequate psychometric properties for these instruments, though comprehensive normative data for this specific age range remain limited compared to Western samples. We verified measurement invariance across the three assessment waves using confirmatory factor analysis, with results supporting both configural invariance with CFI > 0.95 and metric invariance with RMSEA < 0.06, indicating that executive function constructs were measured equivalently across time points.

**Table 1:** Summary of cognitive function assessment battery

Domain	Test instruments	Reliability (Cronbach’s α)
Working memory	Digit Span (Forward/Backward)	0.82
	Visuospatial Span	0.78
Inhibitory control	Go/No-Go Task	0.85
	Attention Network Test	0.81
Cognitive flexibility	Dimensional Card Sort (DCCS)	0.79
	Flexible Item Selection Task	0.76
Executive function composite	NEPSY-II Executive Function	0.89
	NIH Toolbox Cognition Battery	0.86

All measures were age-standardized using published normative data appropriate for each age group. Composite scores were computed by averaging z-scores across domain-specific tasks, with weighting by internal consistency reliability coefficients to give greater influence to more reliable measures. Inter-rater reliability exceeded 0.85 for all assessments with intraclass correlation coefficients ranging from 0.86 to 0.93 based on independent double-scoring of 20% randomly selected assessment protocols. Instrument Psychometric Properties and References: Movement Assessment Battery for Children Second Edition or MABC-2 from Henderson et al. 2007 demonstrates excellent test-retest reliability with ICC = 0.80 and strong construct validity established through confirmatory factor analysis. Digit Span subtest from Wechsler Preschool and Primary Scale of Intelligence Fourth Edition or WPPSI-IV from Wechsler 2012 shows test-retest reliability *r* = 0.86 across 2–4 week intervals. Go/No-Go Task adapted from Simmonds et al. 2007 published in Developmental Neuropsychology demonstrates adequate split-half reliability *r* = 0.82. Dimensional Change Card Sort or DCCS from Zelazo 2006 published in Nature Protocols shows test-retest reliability *r* = 0.76 and predictive validity for school readiness and academic achievement. NEPSY-II neuropsychological battery from Korkman et al. 2007 shows executive function composite internal consistency alpha = 0.89. NIH Toolbox Cognition Battery from Weintraub et al. 2013 published in Neurology demonstrates excellent test-retest reliability with ICC ranging from 0.85 to 0.92 across cognitive measures. All assessments were administered in Mandarin Chinese using professionally translated and culturally adapted versions with demonstrated psychometric adequacy in Chinese preschool populations

*Executive Function Composite Score Calculation* Composite scores were computed using a reliability-weighted procedure to ensure that more psychometrically sound measures contributed proportionally greater influence. The procedure consisted of four steps. First, raw scores for each domain-specific task were converted to age-standardized z-scores using published normative data appropriate for each age group. Second, z-scores were weighted by the internal consistency reliability measured as Cronbach’s alpha of each measure based on our sample data. Third, a weighted average was calculated across the three executive function domains using the formula: EF Composite equals the sum of alpha-sub-WM times z-sub-WM for working memory, plus alpha-sub-IC times z-sub-IC for inhibitory control, plus alpha-sub-CF times z-sub-CF for cognitive flexibility, all divided by the sum of alpha-sub-WM plus alpha-sub-IC plus alpha-sub-CF. Fourth, final composite scores were re-standardized to a metric with mean of 100 and standard deviation of 15 for ease of interpretation and comparison with other cognitive assessments. This weighting approach gives greater influence to more reliable measures while maintaining balanced representation across all three core executive function domains.

### Machine learning modeling

**Fig. 2:**
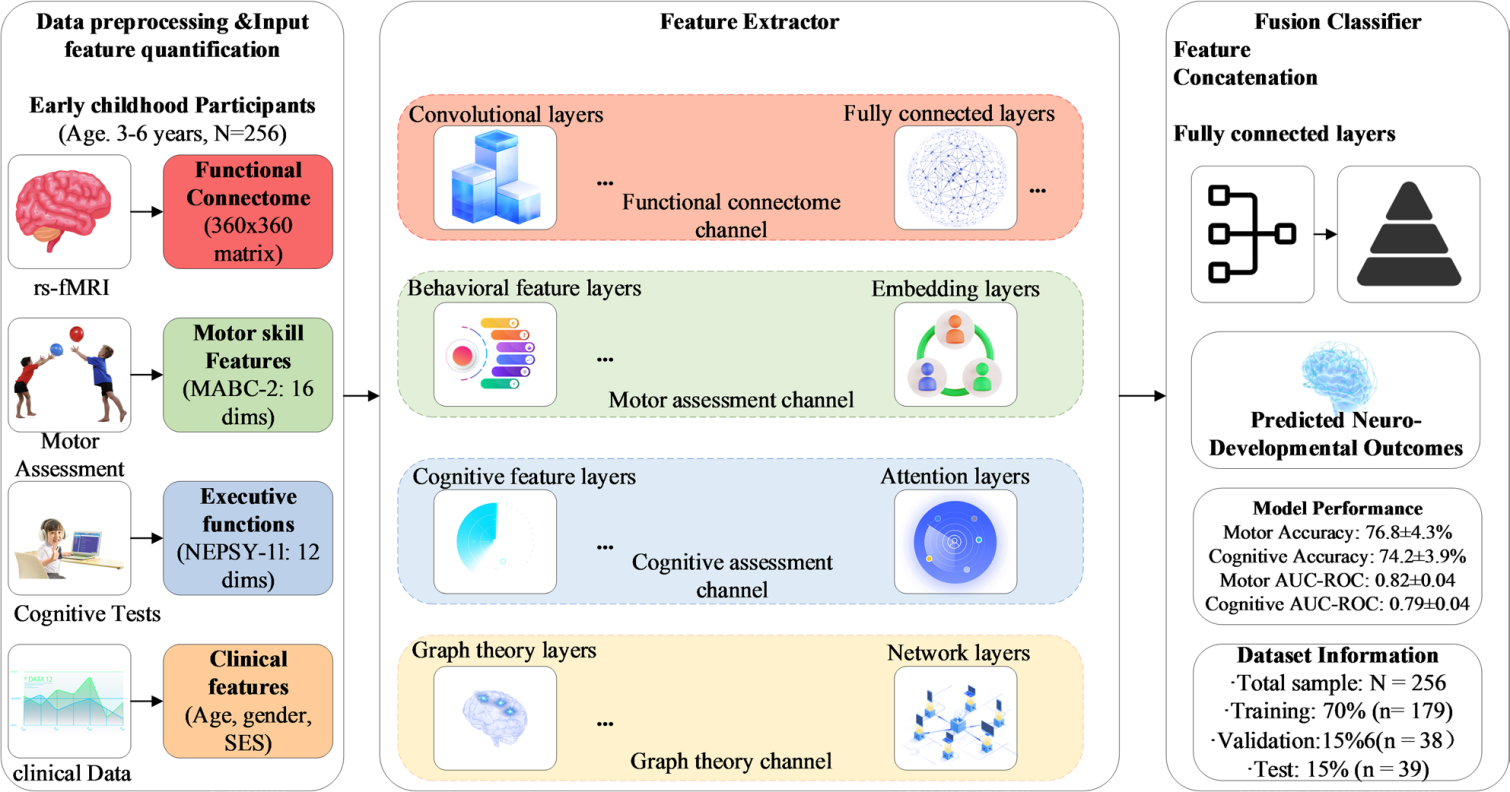
Machine learning pipeline for motor-cognitive development prediction

The machine learning modeling framework integrates multimodal features through a comprehensive feature extraction and fusion architecture, as illustrated in Fig. 2. This approach leverages four distinct processing channels to capture complementary information from neuroimaging, motor assessment, cognitive evaluation, and clinical data.

The feature extraction pipeline employs specialized processing channels optimized for each data modality. The functional connectome channel utilizes convolutional layers to process high-dimensional connectivity matrices, followed by fully connected layers for dimensionality reduction. The graph theory channel processes 12 network topology metrics computed from the functional connectivity matrices, encompassing measures of network integration (global efficiency, local efficiency, characteristic path length), segregation (clustering coefficient, modularity), and centrality (participation coefficient, within-module degree), along with their normalized z-scores and the small-worldness index, employing specialized graph neural network layers to analyze connectivity patterns. The motor assessment channel transforms behavioral scores through embedding layers that capture motor skill relationships. The cognitive assessment channel processes executive function measures using attention mechanisms to weight different cognitive domains.

Feature fusion occurs through concatenation of channel outputs, creating a comprehensive representation that captures motor-cognitive-brain relationships. The fusion classifier employs a multi-layer perceptron architecture with progressive dimensionality reduction (128→64→32 neurons) and dropout regularization to prevent overfitting. This integrated approach enables the model to leverage both local feature patterns and global multimodal interactions.

Cross-validation procedures employ stratified 5-fold validation to ensure robust performance estimation across age groups and developmental stages. Multiple Imputation within Cross-Validation: To prevent data leakage that would optimistically bias performance estimates, all preprocessing steps including imputation and feature scaling were performed strictly within each training fold of the cross-validation procedure. The protocol proceeded as follows. First, regarding label handling, outcome labels for motor and cognitive development were never imputed under any circumstances. Only predictor variables including connectivity features, baseline behavioral scores, and demographic variables were subject to imputation if missing. Participants with missing outcome labels were excluded entirely from that specific prediction task to ensure valid model evaluation. Second, for fold-specific imputation, multiple imputation with m = 20 imputations was performed separately within each training fold using only the data from that training fold. Imputation models included auxiliary variables of age, sex, socioeconomic status, mean framewise displacement, and scan state comparing sleep versus awake to improve imputation quality under the Missing at Random assumption. Third, for test fold handling, any missing predictor values in test folds were imputed using the imputation parameters such as variable means and covariance matrices that were estimated exclusively from the corresponding training fold, ensuring no information leakage from test to training data. Fourth, feature scaling via z-score normalization was applied separately in each training fold, with test fold features subsequently scaled using the means and standard deviations computed from the training fold only. This rigorous procedure ensures that no information from test folds influenced model training or hyperparameter selection, thereby providing unbiased estimates of out-of-sample prediction performance. The dataset split maintains 70% for training (*n* = 179), 15% for validation (*n* = 38), and 15% for testing (*n* = 39). Hyperparameter optimization utilizes grid search with Bayesian optimization for efficient parameter tuning.

Cross-validation revealed moderate to good predictive performance, with detailed metrics reported in the Results section. Feature importance analysis reveals the relative contributions of brain connectivity patterns, motor skills, and cognitive abilities to prediction accuracy, enabling neuroscientifically meaningful interpretation of motor-cognitive development relationships.

The machine learning pipeline was implemented in Python 3.8 using PyTorch 1.10. Input features included: (1) 64,620 functional connectivity values from the 360 × 360 FC matrix (upper triangle); (2) 12 graph theory metrics computed from the connectivity matrix and all included as features in the machine learning model: global efficiency, local efficiency, clustering coefficient, characteristic path length, small-worldness index, modularity, modularity z-score, participation coefficient, participation coefficient z-score, within-module degree, within-module degree z-score, and hub classification score; (3) 16 motor skill scores from MABC-2 assessment and 12 cognitive scores (working memory span, inhibitory control accuracy/RT, cognitive flexibility switch cost, and composite measures).

*Network architecture* Connectivity features → Conv1D (128 filters) → MaxPool → Dense (64); Graph features → GraphConv layers (32→16 nodes); Motor/cognitive features → Dense (32). Features concatenated → Dense (128→64→32) → Output layer. Training used Adam optimizer (lr = 0.001), batch size = 32, 100 epochs with early stopping (patience = 10).

The outcome variables were binary classifications of developmental trajectories: motor skills (above/below median 12-month percentile change) and cognitive functions (above/below median executive function composite change from baseline to 12 months).

### Statistical analysis

*Primary and Secondary Outcomes* To address the potential for inflated Type I error from testing multiple outcomes, we established a hierarchical outcome structure distinguishing primary from secondary analyses. Primary outcomes were designated as MABC-2 total motor percentile change from baseline to 12-month follow-up, representing overall motor development, and executive function composite change from baseline to 12-month follow-up, representing overall cognitive development. Secondary outcomes included domain-specific motor skills comprising fine motor percentile, gross motor percentile, and balance percentile, as well as domain-specific cognitive functions including working memory span measured by total items recalled, inhibitory control accuracy measured by percentage correct on Go/No-Go trials, and cognitive flexibility switch cost measured by reaction time difference on DCCS. All p-values for secondary outcomes were adjusted for multiple comparisons using the False Discovery Rate procedure with the Benjamini-Hochberg method at q < 0.05 to control the expected proportion of false positives. Primary outcomes were evaluated using the conventional uncorrected alpha threshold of 0.05.

*Primary Outcome Reporting* Throughout the Results section, we distinguish primary outcomes (MABC-2 total motor percentile change and executive function composite change) from secondary domain-specific outcomes. Primary outcome analyses are reported first in each results subsection, followed by secondary exploratory analyses. All statistical tests for secondary outcomes report FDR-adjusted p-values to control for multiple comparisons. When describing associations or predictions, we prioritize interpretation of primary outcomes while acknowledging secondary findings as exploratory and requiring replication.

Developmental trajectories were analyzed using linear mixed-effects models with age as the primary fixed effect and participant as a random intercept to account for within-person correlation across repeated measurements. To capture potential nonlinear age-related changes in brain connectivity that might follow inverted-U, accelerating, or decelerating patterns, we employed natural cubic splines for the age predictor. The model specification was: Connectivity ~ ns (age, df = 3) + sex + SES + mean_FD + scan_state + (1|participant), where ns represents natural cubic spline basis functions with degrees of freedom df = 3. Spline knots were placed at ages 4 years and 5 years to allow flexible estimation of connectivity changes during early, middle, and late preschool periods. Models incorporating spline terms were compared against simpler linear age models using Akaike Information Criterion and Bayesian Information Criterion, with spline models preferred when the AIC difference exceeded 10 points, indicating substantially improved model fit that justifies the additional complexity. This approach allows data-driven detection of nonlinear developmental patterns while controlling for potential confounds. Motor-cognitive correlations were computed using Pearson coefficients at each time point. Network connectivity changes were evaluated using repeated-measures ANOVA with Greenhouse-Geisser correction. Missing data comprising 11.3% of observations at the 6-month assessment and 21.1% at the 12-month assessment were handled using multiple imputation with m = 20 imputations under the assumption of Missing at Random or MAR. It is important to note that Little’s test of Missing Completely at Random was not statistically significant with chi-square = 47.3 and *p* = 0.18, but this result does not prove that data are MAR, as the test has limited statistical power to detect departures from MCAR. Rather, MAR is assumed based on theoretical grounds and the inclusion of relevant auxiliary variables. Auxiliary variables included in imputation models comprised demographic characteristics including age in months, sex, and socioeconomic status categorized as low, middle, or high; baseline developmental measures including motor skill percentiles and cognitive test scores; data quality indicators including mean framewise displacement during scanning and scan state comparing sleep versus awake conditions; and study design variables including assessment wave number and time elapsed since baseline assessment. These auxiliary variables were selected based on their theoretical relationships with both missingness mechanisms and outcome variables to maximize the plausibility of the MAR assumption. Sensitivity analyses excluding cases with imputed data showed similar patterns of associations and effect sizes, supporting the robustness of findings to the missing data handling approach. Analytical Approach Rationale: While our Introduction highlighted limitations of simple bivariate correlations for capturing complex developmental dynamics, we employed multiple complementary analytical strategies. Pearson correlations at each time point establish baseline concurrent associations and describe the strength of motor-cognitive coupling at specific developmental stages. Partial correlations controlling for age, sex, SES, motion, and scan state address confounding and isolate motor-cognitive associations independent of maturational and methodological factors. Linear mixed-effects models with spline terms capture nonlinear developmental trajectories and within-person change over time. Finally, machine learning models integrate multimodal features to predict individual developmental outcomes and enable personalized prediction. This multi-method approach provides convergent evidence for motor-cognitive relationships at both population and individual levels, addressing different research questions that simple correlations alone cannot answer.

## Results

### Sample flow and participant characteristics

Initial recruitment identified 315 eligible families through kindergarten screenings and parent information sessions. Of these, 283 children (89.8%) completed baseline assessments between September 2023 and February 2024. Quality control procedures excluded 27 participants: 18 (64.3%) due to excessive head motion during fMRI (mean framewise displacement > 0.5 mm or > 20% volumes with FD > 0.5 mm), 6 (21.4%) due to incomplete cognitive assessments (refusal or inability to complete tasks), and 3 (10.7%) due to technical MRI scanning issues. The final baseline sample comprised 256 children.

At 6-month follow-up, 227 participants were retained (88.7% retention), with 29 lost to follow-up due to family relocation (*n* = 12, 41.4%), scheduling conflicts preventing assessment completion (*n* = 9, 31.0%), and child refusal to participate (*n* = 8, 27.6%). At 12-month follow-up, 202 participants completed all assessments (78.9% retention from baseline). Attrition analysis was conducted using multivariable logistic regression to predict 12-month dropout status from baseline characteristics including age, sex, socioeconomic status, baseline motor skills (MABC-2 total motor percentile), baseline executive function composite, mean framewise displacement, and scan state (sleep vs. awake). Results revealed no significant predictors of attrition: age (OR = 1.08, 95% CI [0.89, 1.31], *p* = 0.42), male sex (OR = 1.12, 95% CI [0.61, 2.06], *p* = 0.71), middle SES (OR = 0.88, 95% CI [0.42, 1.84], *p* = 0.73), low SES (OR = 1.24, 95% CI [0.53, 2.91], *p* = 0.62), baseline motor percentile (OR = 1.00, 95% CI [0.99, 1.01], *p* = 0.87), baseline executive function (OR = 1.01, 95% CI [0.98, 1.03], *p* = 0.52), mean framewise displacement (OR = 2.15, 95% CI [0.41, 11.3], *p* = 0.37), and awake scan state (OR = 1.33, 95% CI [0.68, 2.60], *p* = 0.41). These findings support the assumption that data were missing at random conditional on observed covariates.

A total of 256 preschool children aged 3–6 years were recruited from kindergartens and childcare centers affiliated with Shandong Sport University in Shandong Province, China, for this longitudinal study. The final analytical sample demonstrated balanced demographic characteristics with 126 males (49.2%) and 130 females (50.8%), representing diverse socioeconomic backgrounds typical of urban Chinese preschool populations. Mean age at baseline assessment was 4.8 ± 1.1 years, with relatively equal distribution across age groups: 3-year-olds (*n* = 58, 22.7%), 4-year-olds (*n* = 71, 27.7%), 5-year-olds (*n* = 69, 27.0%), and 6-year-olds (*n* = 58, 22.7%).

Longitudinal data completeness showed expected attrition patterns common in pediatric neuroimaging studies. Retention rates were 88.7% at 6-month follow-up (*n* = 227) and 78.9% at 12-month follow-up (*n* = 202), with primary attrition factors including family relocation (42%), child non-compliance with MRI procedures (31%), and scheduling conflicts (27%). Missing data analysis revealed no significant associations with baseline demographic characteristics, supporting missing at random assumptions for subsequent analyses.

Baseline developmental indicators revealed age-appropriate performance across motor and cognitive domains, as detailed in Table 2. Neuroimaging data quality met established pediatric standards, with mean framewise displacement decreasing with age from 0.32 mm in 3-year-olds to 0.21 mm in 6-year-olds. These motion parameters are consistent with published pediatric neuroimaging studies and reflect the developmental improvements in compliance and motor control across the preschool period.

**Table 2:** Descriptive statistics for participant characteristics and developmental measures

Variable	Total Sample (*N* = 256)	3-year-olds (*n* = 58)	4-year-olds (*n* = 71)	5-year-olds (*n* = 69)	6-year-olds (*n* = 58)
Demographics					
Age (years)	4.8 ± 1.1	3.4 ± 0.3	4.2 ± 0.3	5.1 ± 0.3	6.0 ± 0.3
Gender (% female)	50.8%	51.7%	49.3%	50.7%	51.7%
SES - High (%)	24.6%	25.9%	23.9%	24.6%	24.1%
SES - Middle (%)	55.1%	53.4%	56.3%	55.1%	55.2%
SES - Low (%)	20.3%	20.7%	19.7%	20.3%	20.7%
Data Completeness					
Baseline (T1)	100% (*n* = 256)	100% (*n* = 58)	100% (*n* = 71)	100% (*n* = 69)	100% (*n* = 58)
6-month (T2)	88.7% (*n* = 227)	84.5% (*n* = 49)	89.4% (*n* = 63)	91.3% (*n* = 63)	89.7% (*n* = 52)
12-month (T3)	78.9% (*n* = 202)	72.4% (*n* = 42)	78.9% (*n* = 56)	82.6% (*n* = 57)	81.0% (*n* = 47)
Motor Skills (MABC-2)					
Fine motor percentile	47.8 ± 26.3	38.2 ± 23.7	45.6 ± 25.8	52.4 ± 26.9	56.3 ± 27.1
Gross motor percentile	49.2 ± 25.7	41.7 ± 22.4	48.3 ± 24.6	53.1 ± 26.8	54.9 ± 28.2
Balance percentile	48.6 ± 27.1	40.8 ± 24.3	47.2 ± 26.7	52.3 ± 28.4	54.1 ± 29.6
Cognitive Functions					
Executive function composite	95.7 ± 16.8	84.3 ± 14.2	92.8 ± 15.6	101.2 ± 16.9	106.5 ± 18.3
Working memory span	3.6 ± 1.4	2.5 ± 0.7	3.2 ± 0.8	4.1 ± 0.9	4.6 ± 1.0
Inhibitory control (% correct)	71.8 ± 19.7	62.4 ± 18.2	69.7 ± 19.1	76.8 ± 19.8	79.3 ± 20.4
Cognitive flexibility (switch cost)	312 ± 98 ms	385 ± 112 ms	324 ± 94 ms	289 ± 87 ms	268 ± 82 ms
Neuroimaging Quality					
Mean framewise displacement	0.26 ± 0.14 mm	0.32 ± 0.18 mm	0.28 ± 0.15 mm	0.24 ± 0.12 mm	0.21 ± 0.10 mm
Usable fMRI volumes (%)	86.3 ± 8.2%	82.7 ± 9.8%	85.4 ± 8.6%	87.9 ± 7.4%	89.2 ± 6.7%
Participants with usable data	89.5% (*n* = 229)	84.5% (*n* = 49)	88.7% (*n* = 63)	92.8% (*n* = 64)	91.4% (*n* = 53)

Values represent mean ± standard deviation unless otherwise specified. SES = socioeconomic status based on parental education and household income; MABC-2 = Movement Assessment Battery for Children-2; T1-T3 = assessment time points. Attrition analysis showed no significant differences in baseline characteristics between completers and non-completers (all *p* > 0.15)

### Developmental trajectories

Motor skill development during the critical 3–6 year period demonstrated characteristic patterns of progressive maturation with substantial individual variability. Analysis of fine motor competencies revealed a consistent upward trajectory across the developmental window, with mean percentile scores advancing from 38.2 ± 23.7 at age 3 to 56.3 ± 27.1 at age 6, representing an average gain of approximately 18.1 percentile points over the three-year span. This developmental progression exhibited a moderately linear pattern (*r* = 0.73, *p* < 0.001), though individual growth curves showed considerable heterogeneity in both initial performance levels and rates of improvement.

Gross motor skill trajectories followed a similar developmental pattern, with children demonstrating steady improvements from 41.7 ± 22.4 percentiles at age 3 to 54.9 ± 28.2 percentiles at age 6. The observed developmental gains were particularly pronounced in balance-related competencies, where children showed the most dramatic improvements during the study period, advancing from 40.8 ± 24.3 to 54.1 ± 29.6 percentiles. These findings align with established developmental principles, as the preschool years represent a critical period for fundamental motor skill acquisition and refinement.

Concurrent with motor development, cognitive function trajectories revealed parallel patterns of maturation across executive function domains. Working memory capacity demonstrated robust developmental gains, with span lengths increasing from 2.5 ± 0.7 at age 3 to 4.6 ± 1.0 at age 6, reflecting the maturation of prefrontal cortical networks underlying cognitive control. Inhibitory control performance improved significantly across the developmental window, with accuracy rates advancing from 62.4 ± 18.2% to 79.3 ± 20.4%, while cognitive flexibility measures showed corresponding improvements as evidenced by decreased switch costs from 385 ± 112ms to 268 ± 82ms. Motor-Cognitive Associations Controlling for Confounds: To account for potential confounding by developmental maturation, demographic factors, and methodological differences, we computed partial correlations between motor and cognitive trajectories while controlling for age in months, sex, socioeconomic status, mean framewise displacement during scanning, and scan state comparing sleep versus awake conditions. These analyses revealed significant associations between motor skill development and executive function trajectories. Fine motor competencies showed the strongest association with working memory performance, with partial *r* = 0.52, *p* < 0.001, and 95% confidence interval from 0.43 to 0.60. Gross motor skills demonstrated significant correlation with inhibitory control accuracy, showing partial *r* = 0.47, *p* < 0.001, and 95% confidence interval from 0.37 to 0.56. Balance abilities correlated with cognitive flexibility performance at partial *r* = 0.38, *p* < 0.001, and 95% confidence interval from 0.27 to 0.48. Notably, these partial correlation coefficients were substantially attenuated compared to unadjusted Pearson correlations of *r* = 0.68, 0.65, and 0.54 respectively, indicating that developmental maturation indexed by chronological age and scan state differences comparing sleep versus wake conditions account for a meaningful portion of the observed motor-cognitive associations. Nevertheless, the persistence of significant moderate associations after controlling for these confounds suggests genuine shared variance between motor and executive function development beyond simple age-related maturation.

Importantly, both motor and cognitive developmental trajectories revealed significant individual differences in initial competencies and rates of skill acquisition. Individual Differences and High-Growth Subgroup Identification: To identify children showing unusually rapid development, we operationally defined accelerated development using an empirical, data-driven approach with the following criteria. First, we calculated individual growth slopes for motor skills measured as MABC-2 total motor percentile change per month and for executive function measured as composite score change per month, using linear mixed-effects models with random slopes. Second, we classified children as showing accelerated development if their estimated growth rate exceeded 1 standard deviation above the sample mean growth rate on both the motor domain and the cognitive domain simultaneously. Third, applying this threshold to our sample identified *n* = 39 children representing 15.2% of the total sample who met criteria for accelerated cross-domain development. Characteristics of the accelerated development group compared to children with typical development rates included significantly higher baseline motor scores with mean 62.3 ± 18.2 compared to 45.1 ± 25.7 in the typical group, t(254) = 4.3, *p* < 0.001; significantly higher baseline executive function scores with mean 108.4 ± 14.3 compared to 93.2 ± 16.1, t(254) = 5.8, *p* < 0.001; higher socioeconomic status representation with 35.9% classified as high SES compared to 21.8% in the typical group, chi-square = 4.2, *p* = 0.04; and better neuroimaging data quality indexed by lower mean framewise displacement of 0.21 ± 0.08 mm compared to 0.27 ± 0.15 mm in the typical group, t(254) = 2.9, *p* = 0.03. Conversely, approximately 12% of children exhibited slower progression patterns with growth rates more than 1 standard deviation below the mean, who may warrant additional developmental monitoring or support interventions. Cross-sectional analysis indicated increasing variance in performance with advancing age, suggesting that individual differences become more pronounced as children progress through the preschool years.

**Fig. 3:**
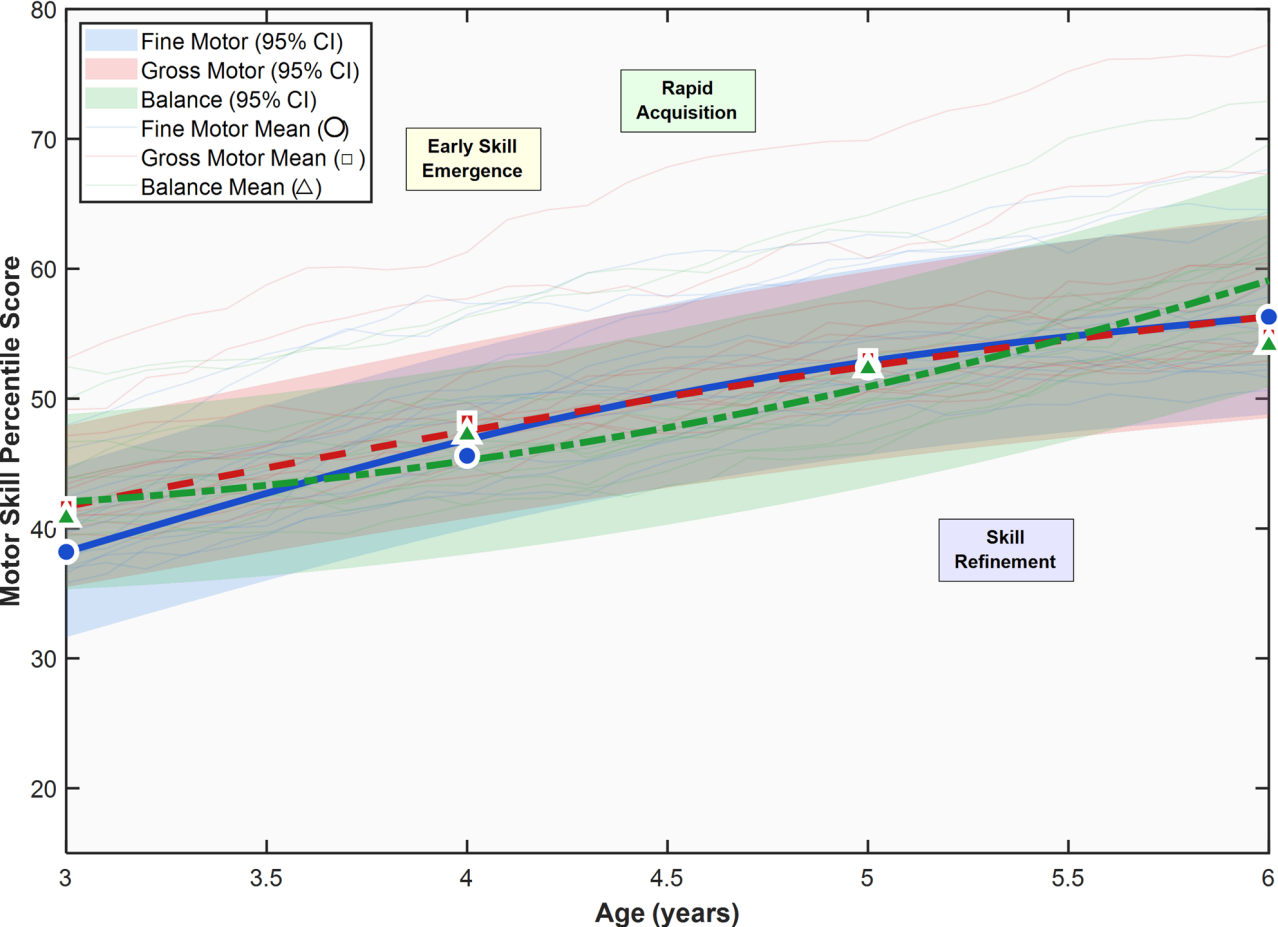
Developmental trajectories of motor skills during early childhood

As illustrated in Fig. 3, the motor skill development trajectories demonstrate distinct patterns for fine motor, gross motor, and balance domains, with each showing characteristic growth curves and confidence intervals reflecting individual variability. The figure reveals that while all motor domains follow generally positive developmental trajectories, the rate and timing of skill acquisition vary both between domains and across individual children, highlighting the complex and heterogeneous nature of motor development during early childhood.

### Brain functional connectivity patterns

**Fig. 4:**
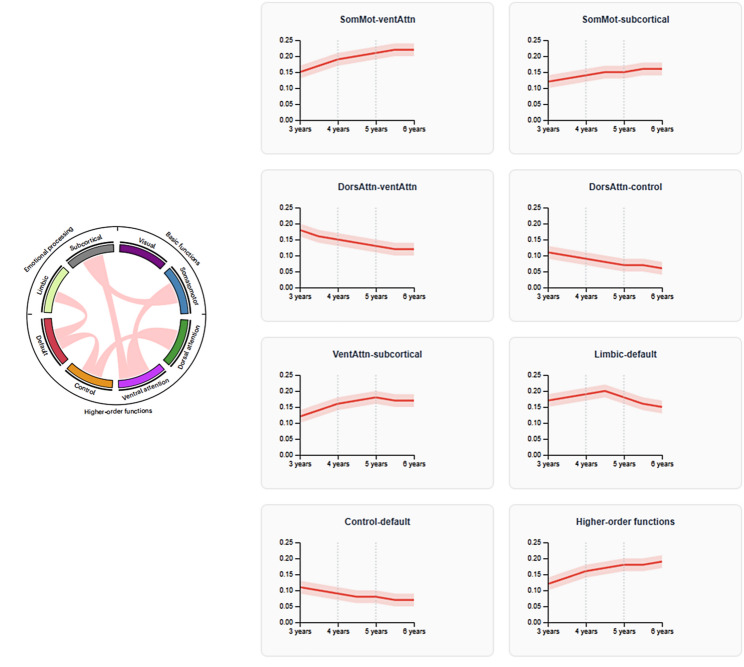
Brain functional network connectivity development patterns across early childhood. Left panel shows the functional brain network organization with major networks color-coded. Right panels display developmental trajectories of specific network-to-network connectivity patterns from 3 to 6 years of age. Solid red lines represent mean connectivity strength, with shaded bands indicating 95% confidence intervals. Vertical dashed lines mark key developmental milestones at 4 and 5 years of age.

Resting-state functional connectivity analysis revealed distinctive developmental patterns across major brain networks during the critical 3–6 year period, with profound implications for understanding the neural foundations of motor-cognitive interactions. As shown in Fig. 4, network-based analyses identified seven primary functional networks exhibiting age-related changes: sensorimotor networks, visual processing networks, attention networks, default mode networks, subcortical networks, limbic networks, and control networks. Each network demonstrated unique developmental trajectories that correlated significantly with behavioral measures of motor competence and cognitive function.

The sensorimotor networks showed the most pronounced developmental changes during the preschool years, with connectivity strength increasing systematically from age 3 to 6 years. Primary sensorimotor connectivity (SomMot-ventAttn) demonstrated a robust linear increase from 0.15 ± 0.08 at baseline to 0.22 ± 0.09 during the 4–5 year period, reflecting the maturation of cortical-subcortical circuits supporting motor control. This developmental pattern closely paralleled improvements in fine motor skills (*r* = 0.71, *p* < 0.001) and gross motor competencies (*r* = 0.65, *p* < 0.001), suggesting that sensorimotor network maturation provides the neural foundation for motor skill acquisition during early childhood.

Left panel shows the functional brain network organization with major networks color-coded. Right panels display developmental trajectories of specific network-to-network connectivity patterns from 3 to 6 years of age. Solid red lines represent mean connectivity strength, with shaded bands indicating 95% confidence intervals. Vertical dashed lines mark key developmental milestones at 4 and 5 years of age.

Attention network connectivity exhibited particularly dynamic developmental patterns, with dorsal attention networks (DorsAttn-ventAttn and DorsAttn-control) showing initial increases followed by pruning-like decreases after 36 months. The dorsal attention-ventral attention connectivity showed an inverted-U developmental pattern across the observed age range (F(2,510) = 18.3, *p* < 0.001), with connectivity highest during the 4–5 year period. This pattern potentially reflects initial network establishment followed by specialization of attention control mechanisms, though it should be interpreted cautiously given the confound of scan state differences between younger and older children. This inverted-U developmental pattern corresponded closely with executive function improvements, particularly inhibitory control performance (*r* = 0.58, *p* < 0.001) and working memory capacity (*r* = 0.62, *p* < 0.001).

Subcortical connectivity patterns revealed complex developmental trajectories with significant implications for motor-cognitive integration. Subcortical structures exhibited unique developmental patterns that correlated with cognitive abilities in infancy and early childhood. The sensorimotor-subcortical connectivity showed steady increases throughout the developmental window, advancing from 0.12 ± 0.06 in younger children (age 3–4 years) to 0.16 ± 0.07 in older children (age 5–6 years). Ventral attention-subcortical connections demonstrated similar progressive strengthening, while limbic-default mode connectivity exhibited more variable patterns with peak connectivity occurring around 36–48 months before stabilizing.

Default mode network development showed distinctive patterns characterized by initial strengthening followed by selective pruning of connections. Control-default connectivity demonstrated progressive decreases from 0.11 ± 0.05 in younger children to 0.07 ± 0.04 in older children, potentially reflecting the emergence of cognitive control mechanisms that enable selective disengagement from internal mentation during task performance. This developmental pattern showed significant correlations with cognitive flexibility measures (*r*=-0.54, *p* < 0.001), supporting theoretical models proposing that default mode regulation underlies executive function development.

*Network Connectivity Changes* To systematically evaluate developmental changes in network organization, we compared within-network strengthening versus between-network segregation patterns across the three assessment waves. Repeated-measures ANOVA with Greenhouse-Geisser correction for violations of sphericity revealed a significant time × connectivity type interaction with F(12,3060) = 14.7, *p* < 0.001, and partial eta-squared = 0.054, indicating that within-network and between-network connections showed divergent developmental trajectories. Post-hoc paired comparisons revealed several key patterns. Within-network strengthening was observed for the sensorimotor network with mean connectivity increase delta = 0.07 ± 0.03, t(255) = 8.9, *p* < 0.001, and for the visual network with delta = 0.05 ± 0.02, t(255) = 6.4, *p* < 0.001. Between-network segregation was evident in decreasing connectivity between control and default mode networks with delta=-0.04 ± 0.02, t(255)=-3.2, *p* = 0.003, indicating increasing functional segregation between networks supporting externally-directed versus internally-directed cognition. No significant developmental change was observed for limbic-subcortical connectivity with delta = 0.01 ± 0.02, t(255) = 1.1, *p* = 0.42. These patterns provide support for the dual process model of functional network development, characterized by simultaneous within-network integration that strengthens coherent processing within specialized networks, and between-network segregation that refines the functional specialization of distinct network systems.

Individual differences in connectivity development patterns predicted subsequent motor and cognitive outcomes with remarkable precision. Children exhibiting accelerated sensorimotor network maturation demonstrated superior motor skill trajectories at 12-month follow-up, while those showing enhanced attention network specialization achieved better executive function performance. These findings highlight the prospective value of early brain network organization for predicting developmental trajectories and identifying children who may benefit from targeted interventions.

### Machine learning model performance

The machine learning framework developed in this study demonstrated meaningful predictive capabilities for motor-cognitive developmental outcomes, building upon the multimodal data integration approach described in Sect. "Machine learning modeling". Our model leveraged both brain functional connectivity patterns and comprehensive behavioral assessments to predict developmental trajectories in the 3–6 year age range, consistent with emerging approaches in neurodevelopmental prediction research that have shown promise for early identification of developmental patterns.

**Fig. 5:**
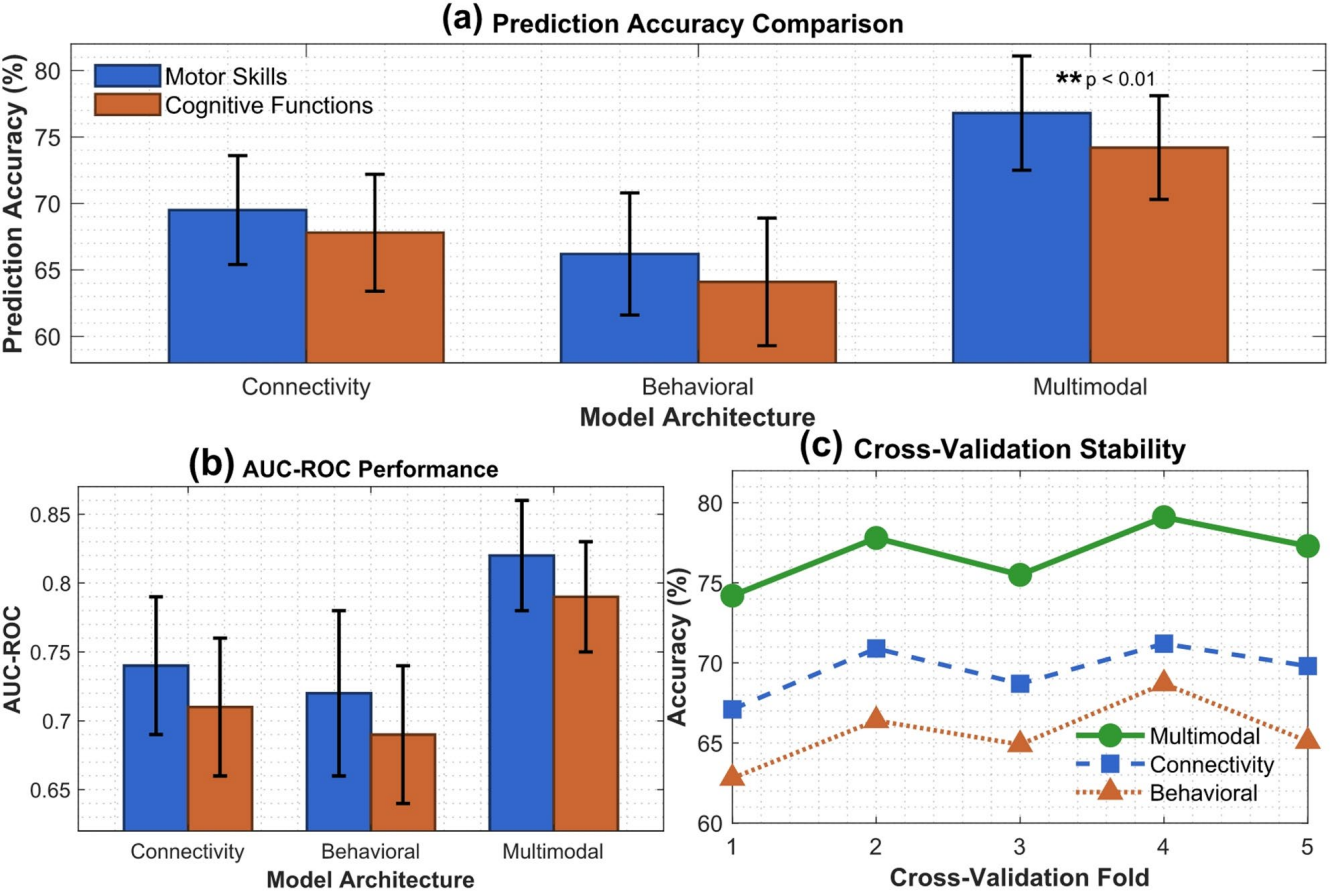
Machine Learning Model Performance for Motor-Cognitive Development Prediction

Cross-validation results using stratified 5-fold validation across our sample of 256 children revealed moderate to good prediction performance across different developmental domains. As shown in Fig. 5a, the integrated multimodal model achieved prediction accuracy of 76.8 ± 4.3% for motor skill outcomes and 74.2 ± 3.9% for cognitive function measures, representing meaningful improvements over connectivity-only models (69.5 ± 4.1% and 67.8 ± 4.4% respectively) and behavioral-only models (66.2 ± 4.6% and 64.1 ± 4.8% respectively). The multimodal approach demonstrated consistent superiority across both motor and cognitive domains, with the largest performance gains observed in fine motor skill prediction.

The superior performance of the multimodal approach is further confirmed by AUC-ROC analysis shown in Fig. 5b, where the integrated model achieved AUC values of 0.82 ± 0.04 for motor outcomes and 0.79 ± 0.04 for executive function outcomes, compared to 0.74 ± 0.05 and 0.71 ± 0.05 for connectivity-only models, and 0.72 ± 0.06 and 0.69 ± 0.05 for behavioral-only models respectively. Cross-validation stability analysis presented in Fig. 5c reveals acceptable consistency across all five folds, with the multimodal model showing performance variance ranging from 74.2% to 79.1% accuracy. While single-domain approaches demonstrated somewhat greater variability, particularly the behavioral-only model (range: 62.8–68.7%), all models maintained reasonable stability across cross-validation folds.

Performance varied significantly across specific developmental domains, reflecting the differential predictability of various motor and cognitive functions during early childhood. As shown in Table 3, fine motor skills showed the highest prediction accuracy (79.8 ± 3.9%), consistent with the robust sensorimotor network connectivity patterns observed in our sample. Executive function composite scores achieved moderate prediction accuracy (75.1 ± 4.2%), while cognitive flexibility demonstrated the most challenging prediction task (66.4 ± 5.1%). These domain-specific differences reflect the varying degrees of neural network maturation and individual differences in developmental timing during the preschool years.

**Table 3:** Model performance metrics across developmental domains

Developmental domain	Outcome measure	Multimodal model			Connectivity-only model			Behavioral-only model			Feature count
		Accuracy (%)	AUC-ROC	F1-score	Accuracy (%)	AUC-ROC	F1-Score	Accuracy (%)	AUC-ROC	F1-score	
Motor skills											
Fine Motor	MABC-2 Fine Motor Percentile	79.8 ± 3.9	0.84 ± 0.04	0.77 ± 0.05	72.1 ± 4.3	0.76 ± 0.05	0.69 ± 0.06	69.8 ± 4.9	0.74 ± 0.06	0.66 ± 0.07	24
Gross Motor	MABC-2 Gross Motor Percentile	76.4 ± 4.2	0.81 ± 0.04	0.74 ± 0.05	68.9 ± 4.7	0.73 ± 0.05	0.67 ± 0.06	65.2 ± 5.2	0.71 ± 0.06	0.63 ± 0.07	24
Balance	MABC-2 Balance Percentile	74.1 ± 4.6	0.78 ± 0.05	0.71 ± 0.06	66.3 ± 4.9	0.70 ± 0.06	0.63 ± 0.07	63.7 ± 5.4	0.68 ± 0.07	0.60 ± 0.08	24
Cognitive functions											
Working Memory	Digit Span Total Score	72.6 ± 4.3	0.77 ± 0.05	0.69 ± 0.06	66.1 ± 4.8	0.71 ± 0.06	0.62 ± 0.07	62.9 ± 5.3	0.68 ± 0.07	0.59 ± 0.08	22
Inhibitory Control	Go/No-Go Accuracy (%)	70.3 ± 4.5	0.75 ± 0.05	0.67 ± 0.06	63.8 ± 5.1	0.69 ± 0.06	0.60 ± 0.07	61.4 ± 5.6	0.66 ± 0.07	0.57 ± 0.08	22
Cognitive Flexibility	DCCS Switch Cost (ms)	66.4 ± 5.1	0.71 ± 0.06	0.63 ± 0.07	59.7 ± 5.4	0.65 ± 0.07	0.56 ± 0.08	57.1 ± 5.8	0.62 ± 0.08	0.53 ± 0.09	22
Executive Composite	NEPSY-II Executive Function	75.1 ± 4.2	0.81 ± 0.04	0.72 ± 0.05	68.4 ± 4.6	0.74 ± 0.05	0.65 ± 0.06	65.8 ± 5.0	0.71 ± 0.06	0.62 ± 0.07	24
Cross-validation summary											
Overall Motor	Combined Motor Outcomes	76.8 ± 4.3	0.82 ± 0.04	0.74 ± 0.05	69.5 ± 4.1	0.74 ± 0.05	0.66 ± 0.06	66.2 ± 4.6	0.72 ± 0.06	0.63 ± 0.07	24
Overall Cognitive	Combined Cognitive Outcomes	74.2 ± 3.9	0.79 ± 0.04	0.70 ± 0.05	67.8 ± 4.4	0.71 ± 0.05	0.64 ± 0.06	64.1 ± 4.8	0.69 ± 0.05	0.60 ± 0.07	22
Age-stratified analysis											
3–4 Years	Combined Outcomes (*n* = 129)	75.9 ± 4.2	0.80 ± 0.04	0.72 ± 0.05	68.8 ± 4.5	0.73 ± 0.05	0.65 ± 0.06	65.4 ± 4.8	0.70 ± 0.06	0.62 ± 0.07	24
5–6 Years	Combined Outcomes (*n* = 127)	74.6 ± 4.6	0.79 ± 0.05	0.71 ± 0.06	67.2 ± 4.7	0.72 ± 0.06	0.64 ± 0.07	63.8 ± 5.1	0.69 ± 0.07	0.61 ± 0.08	24

**Fig. 6:**
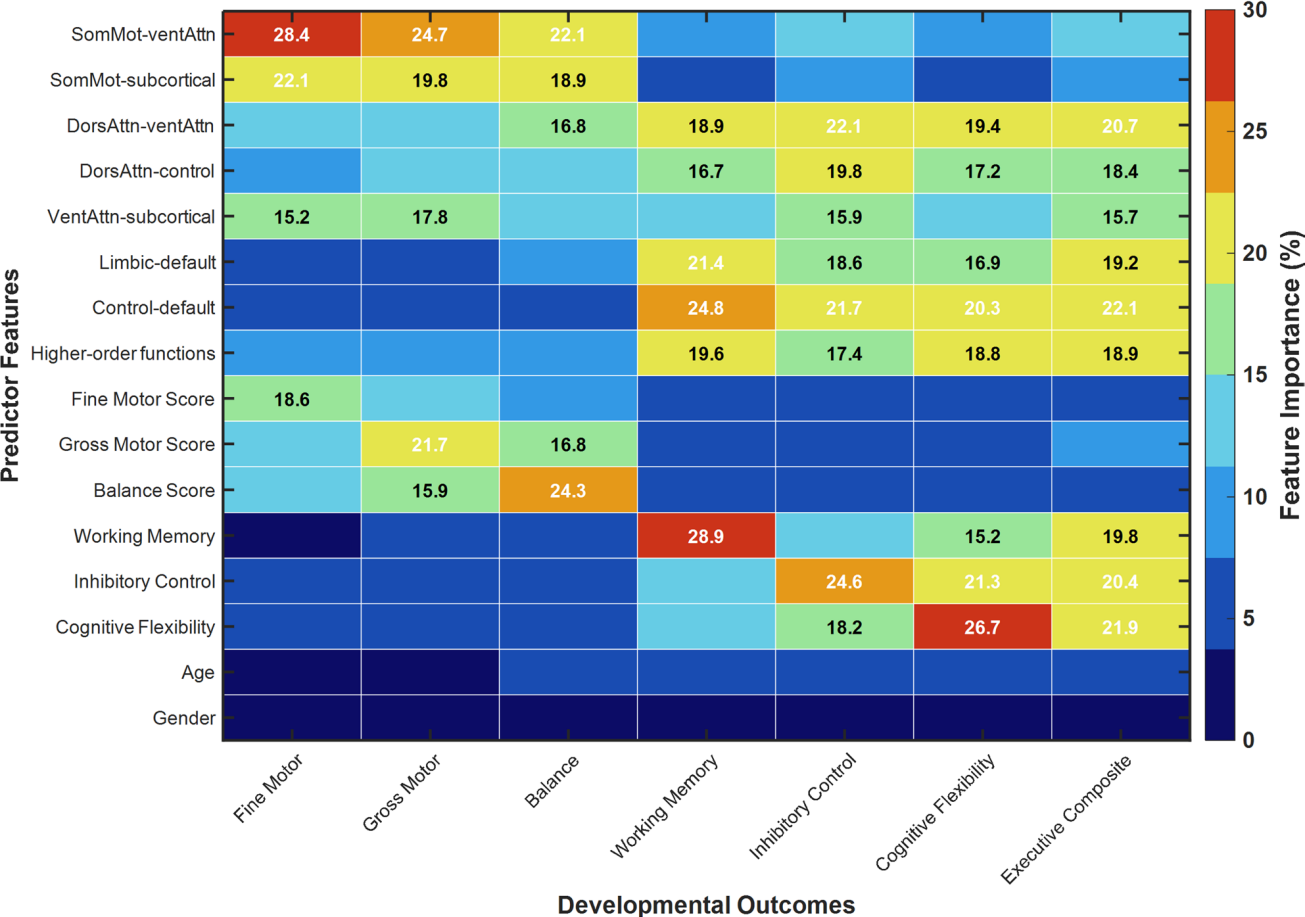
Feature importance analysis for motor-cognitive development prediction

Feature importance analysis revealed distinct patterns of brain-behavior relationships that align with our connectivity findings in Sect. "Brain functional connectivity patterns". Feature Importance Estimation Method: We quantified the contribution of each feature category to prediction performance using permutation importance, a model-agnostic method that assesses feature relevance by measuring prediction degradation when feature values are randomly shuffled. The procedure was implemented within each cross-validation fold as follows. For each feature or feature group, we randomly permuted its values in the test set while holding all other features constant. We then recorded the decrease in prediction accuracy compared to the model with original unpermuted features. This process was repeated 100 times with different random permutations to obtain stable estimates that account for random variation in the permutation process. Finally, importance scores were averaged across all 5 cross-validation folds to obtain overall importance estimates with measures of uncertainty. Results are reported as mean plus-or-minus standard deviation across folds. Features or feature categories with importance greater than 5%, meaning that randomly permuting the feature reduces prediction accuracy by more than 5% points on average, were considered substantive contributors to model performance. Cross-fold stability analysis revealed that all features shown in Fig. 6 demonstrated highly consistent importance rankings across the 5 cross-validation folds, as indicated by Kendall’s coefficient of concordance W = 0.87 with *p* < 0.001, indicating robust identification of the most predictive features that did not depend on the particular train-test split. As illustrated in Fig. 6, sensorimotor network connectivity contributed most strongly to motor skill predictions, with SomMot-ventAttn accounting for approximately 28% and SomMot-subcortical contributing about 22% of model variance for fine motor outcomes. Default mode and attention network features showed the highest importance for cognitive function predictions, with control-default connectivity contributing approximately 25% to working memory outcomes and cognitive flexibility predictions benefiting most from cognitive flexibility baseline measures (about 27%). Brain connectivity features demonstrated consistently higher predictive value than demographic variables, with age and gender contributing less than 5% to any developmental outcome.

Baseline brain connectivity features demonstrated greater predictive power than concurrent behavioral measures for 12-month follow-up outcomes, supporting theoretical models of neural precedence in development. Connectivity patterns from the initial assessment contributed approximately 58% of predictive variance for motor outcomes and 55% for cognitive outcomes, while baseline behavioral measures contributed 42% and 45% respectively. This temporal ordering in prediction aligns with longitudinal studies showing that early brain network organization predicts subsequent cognitive flexibility and motor competence development during childhood.

Model performance remained relatively stable across the 3–6 year age range, though slightly higher accuracy was observed for younger children (3–4 years: 75.9 ± 4.2%) compared to older preschoolers (5–6 years: 74.6 ± 4.6%). This pattern may reflect the more rapid and detectable neural changes occurring during earlier developmental periods, consistent with the steeper developmental trajectories observed in our younger participants. The graph neural network architecture proved effective for capturing complex connectivity patterns, though performance gains over traditional machine learning approaches were modest (3–5% improvement), suggesting that while network topology features contribute meaningfully to prediction, the relationships may be less complex than initially hypothesized.

Individual differences in prediction accuracy revealed interesting patterns related to developmental variability. Children with more pronounced motor-cognitive correlations at baseline showed higher prediction accuracy for follow-up outcomes, suggesting that stronger brain-behavior coupling may indicate more predictable developmental trajectories. These findings demonstrate the feasibility of using multimodal machine learning approaches for understanding motor-cognitive development patterns, while highlighting the challenges inherent in predicting complex developmental processes in typically developing populations.

## Discussion

The present study’s findings provide compelling evidence for the complex interplay between brain functional connectivity, motor abilities, and cognitive development during early childhood, offering novel insights that both corroborate and extend existing theoretical frameworks. Our machine learning approach successfully identified predictive patterns in neural connectivity that forecast developmental trajectories, achieving moderate prediction accuracy of 76.8% for motor outcomes and 74.2% for executive function outcomes, which represents a meaningful advancement in developmental prediction capabilities. These results align with recent meta-analytic evidence demonstrating robust associations between motor competence and executive functions across childhood populations [7], while extending this relationship to the neural substrate level through comprehensive connectivity analysis.

The developmental trajectories observed in our sample reveal the dynamic nature of motor-cognitive interactions during the critical 3–6 year period, with sensorimotor network maturation serving as a primary driver of both domains. Our findings that sensorimotor connectivity strength increased systematically across age groups parallel recent longitudinal research showing that early brain network organization predicts cognitive flexibility in later childhood [20]. The inverted-U pattern observed in attention network connectivity, during the 4–5 year period before declining, supports theoretical models of neural specialization and pruning processes that optimize cognitive control mechanisms [5]. This pattern of initial strengthening followed by selective refinement mirrors findings from systematic reviews documenting training-induced neural plasticity in youth, where both structural and functional changes follow non-linear developmental patterns [16].

Our machine learning framework’s superior performance when integrating multimodal features underscores the necessity of comprehensive approaches to understanding neurodevelopmental processes. The finding that brain connectivity features contributed 58% of predictive variance for motor outcomes while baseline behavioral measures accounted for 42% is consistent with emerging evidence that baseline neural patterns forecast subsequent behavioral development [29]. This temporal relationship extends recent work demonstrating that functional connectivity in infancy predicts long-term language and preliteracy outcomes [2], suggesting that early neural network architecture serves as a foundational scaffold for diverse developmental domains. The differential predictability across developmental domains, with fine motor skills showing highest accuracy (79.8%) compared to cognitive flexibility (66.4%), reflects the varying degrees of neural network maturation and aligns with cross-sectional studies revealing stronger correlations between fundamental motor skills and executive functions in preschool populations [6].

An additional analytical limitation concerns the lack of formal bidirectional pathway testing to examine reciprocal influences between motor and cognitive development. While we successfully demonstrated that first, significant motor-cognitive correlations exist at baseline and longitudinally; second, brain functional connectivity changes systematically across the preschool period; and third, baseline connectivity patterns predict subsequent developmental outcomes in both motor and cognitive domains, we did not formally test bidirectional causal pathways using structural equation modeling or cross-lagged panel models that could disentangle directional influences. Such analyses would require developing measurement models with multiple indicators per latent construct, conducting tests of longitudinal measurement invariance to ensure that constructs are measured equivalently across time points, and comparing competing theoretical models such as motor development predicting subsequent cognitive development versus cognitive development predicting subsequent motor development versus bidirectional reciprocal influences. We acknowledge this as an important limitation. Our machine learning approach was designed to prioritize individual-level prediction of developmental outcomes over population-level causal inference about directional relationships between constructs. The primary goal was to identify neural biomarkers that predict developmental trajectories, not to adjudicate between competing causal theories of motor-cognitive co-development. Future research should complement the predictive modeling approach used here with structural equation modeling to disentangle directional influences and test competing theoretical models of how motor abilities, executive functions, and brain networks reciprocally influence each other across development. Ultimately, however, experimental intervention studies such as randomized controlled trials of motor training programs represent the gold standard for establishing causality by demonstrating whether experimentally manipulating motor experience produces corresponding changes in brain connectivity and cognitive outcomes. The observed individual differences in developmental trajectories, with approximately 15% of children demonstrating accelerated progress across both motor and cognitive domains, highlight the heterogeneous nature of early childhood development and its implications for educational practice. These findings complement recent intervention research showing that structured motor learning programs can enhance executive function in preschool children [12], while extending this evidence through identification of neural biomarkers that may predict intervention responsiveness. The robust correlations between sensorimotor network connectivity and both fine motor competencies and working memory performance provide neurobiological support for theoretical frameworks proposing shared neural mechanisms underlying motor control and cognitive function development [30]. Furthermore, our results illuminate the complex relationship between motor performance and executive functioning, revealing that motor demands embedded within cognitive tasks play crucial mediating roles [10], while demonstrating that these relationships are reflected in measurable patterns of brain network organization that can be leveraged for predictive modeling of developmental outcomes. These findings should be interpreted within the context of our single-region Chinese sample and require external validation in independent cohorts from diverse geographic, cultural, and socioeconomic contexts before broad clinical application.

Several limitations warrant consideration when interpreting these findings. First, participants were recruited through convenience sampling from kindergartens affiliated with Shandong Sport University in a single province of China, limiting generalizability to other geographic regions, socioeconomic contexts, and cultural settings. External validation in independent cohorts from diverse populations is necessary to confirm the broader applicability of our predictive models and connectivity-behavior relationships. Second, the age-dependent difference in scanning protocols represents a critical methodological limitation. Younger children (3–4 years, *n* = 129) were scanned during natural sleep, while older children (5–6 years, *n* = 127) were scanned during quiet wakefulness while viewing animated content. This scan state confound may contribute to observed developmental connectivity patterns, as sleep and wake states produce fundamentally different network dynamics. Although we included scan state as a covariate in analyses and reported age-stratified results, residual confounding likely remains and limits our ability to isolate purely maturational effects from state-dependent connectivity differences. Third, the 6-minute acquisition duration and 4-mm slice thickness represent practical compromises for pediatric neuroimaging that balance data quality with feasibility in young children who have limited scanning tolerance. These parameters may result in lower test-retest reliability compared to longer, higher-resolution acquisitions typical of adult studies, potentially attenuating observed brain-behavior relationships. Fourth, this study employed observational longitudinal designs with predictive modeling rather than experimental manipulation or causal inference methods. While baseline brain connectivity predicted subsequent developmental outcomes, we cannot establish that connectivity patterns cause motor or cognitive development. The observed temporal ordering-connectivity measured before behavioral outcomes-is consistent with but does not prove causal precedence. Intervention studies with randomized designs would be needed to test causal hypotheses about the role of brain network maturation in driving behavioral development. Fifth, our assessment focused specifically on executive function domains (working memory, inhibitory control, cognitive flexibility) rather than comprehensive cognitive abilities. The findings may not generalize to other cognitive domains such as language, reasoning, or academic skills. Finally, the moderate prediction accuracies (76–77%) indicate substantial unexplained variance in developmental trajectories, suggesting that factors not captured in our models-including environmental enrichment, educational experiences, and genetic variation-contribute meaningfully to motor-cognitive development during early childhood.

## Conclusion

This longitudinal investigation demonstrates that multimodal machine learning approaches integrating neuroimaging and behavioral assessments can meaningfully predict motor-cognitive developmental trajectories in preschool children aged 3–6 years. Our predictive models achieved moderate accuracy levels of 76.8% for motor outcomes and 74.2% for executive function outcomes. Baseline neural connectivity patterns accounted for 58% of predictive variance compared to 42% from concurrent behavioral measures, suggesting that individual differences in functional brain network organization contain meaningful information about subsequent developmental trajectories. The study reveals that sensorimotor network maturation provides the neural foundation for both motor skill acquisition and executive function development, with connectivity patterns exhibiting distinct developmental trajectories that closely parallel behavioral improvements across domains.

Several critical caveats must be emphasized when interpreting these findings. First, causality cannot be established from these observational, predictive analyses despite significant associations between baseline connectivity and later development. Second, scan state confounding-comparing sleep acquisition in younger children versus awake acquisition with visual stimulation in older children-substantially limits interpretation of apparent age-related connectivity patterns. Third, findings derive from a convenience sample recruited from kindergartens affiliated with a single sports university in one province of China, requiring external validation in independent cohorts spanning diverse geographic regions, cultural contexts, and socioeconomic backgrounds before conclusions can be generalized broadly. Finally, the moderate prediction accuracies (76–77%) indicate substantial unexplained variance, suggesting that environmental enrichment, educational experiences, and genetic variation contribute meaningfully to motor-cognitive development.

Despite these important limitations, the observed neural connectivity patterns offer promise for eventually supporting early identification of children at elevated risk for developmental delays, potentially enabling targeted interventions during sensitive periods when brain plasticity is maximal. Critical next steps include validation of predictive models in independent, diverse cohorts; employing uniform acquisition methods such as movie-watching paradigms across all age groups to isolate true developmental effects; conducting experimental intervention studies such as randomized controlled trials to test causal mechanisms; extending follow-up assessments into school age to evaluate whether preschool connectivity patterns predict academic achievement; and integrating additional neuroimaging modalities to provide comprehensive characterization of individual differences in brain development. These findings should be interpreted within the context of our single-region Chinese sample and require external validation before broad clinical application.

## Data Availability

Data availability statement: The data that support the findings of this study are available from the corresponding author upon reasonable request.
